# Evolutionary dynamics of the chloroplast genome in *Daphne* (Thymelaeaceae): comparative analysis with related genera and insights into phylogenetics

**DOI:** 10.1002/2211-5463.70143

**Published:** 2025-10-16

**Authors:** Abdullah, Hui Li, Rushan Yan, Chengyu Chen, Madiha Islam, Abdul Majid, Ibrar Ahmed, Parviz Heidari, Xiaoxuan Tian

**Affiliations:** ^1^ State Key Laboratory of Chinese Medicine Modernization Tianjin University of Traditional Chinese Medicine China; ^2^ Haihe Laboratory of Modern Chinese Medicine Tianjin China; ^3^ Department of Biotechnology and Genetic Engineering Hazara University Mansehra Pakistan; ^4^ Department of Botany Hazara University Mansehra Pakistan; ^5^ Alpha Genomics Private Limited Islamabad Pakistan; ^6^ Microbiological Analysis Team, Group for Biometrology Korea Research Institute of Standards and Science (KRISS) Daejeon Korea; ^7^ Faculty of Agriculture Shahrood University of Technology Iran

**Keywords:** chloroplast, Daphne, Gyrinops, phylogeny, Thymelaeaceae, Wikstroemia

## Abstract

The family Thymelaeaceae Juss., includes numerous botanically and medicinally important species, yet its taxonomy, particularly within the genus *Daphne* Tourn. ex L., remains unresolved. In the present study, we sequenced and *de novo* assembled the chloroplast (cp) genome of *Daphne mucronata* Royle from Pakistan and assembled cp genomes of four additional *Daphne* species, one *Dirca* L. species and two *Thymelaea* Mill. species using public raw data. Comparative analyses across 14 genera, including 17 *Daphne* species, revealed substantial structural variation in cp genomes. The cp genomes contained 111–112 unique genes, including 77–78 protein‐coding genes, 30 tRNA genes and four rRNA genes. When considering duplicated copies in the inverted repeat regions, the total gene count ranged from 128 to 142 (83–96 protein‐coding genes, 37–38 tRNA genes and eight rRNA genes). Variability in total gene number was primarily driven by inverted repeat contraction and expansion and pseudogenization of some genes. *ndhF* was pseudogenized in seven *Daphne* species, whereas *ndhI* and *ndhG* were pseudogenized in one species each. Two large inversion events were detected, involving 19 and 40 genes, respectively; the first inversion was synapomorphic and provided significant phylogenetic signal. Phylogenetic analysis of shared protein‐coding sequences from 74 species (87 individuals) recovered *Daphne* as a strongly supported monophyletic clade when taxa previously proposed for exclusion from the genus were omitted, whereas *Wikstroemia* Endl. and *Gyrinops* Gaertn. exhibited paraphyly. This study expands the cp genome resources for Thymelaeaceae and provides new insights into the structural evolution of cp genome.

## Graphical abstract

Comparative analysis of chloroplast genomes from 14 genera of Thymelaeaceae revealed variation in gene content, ranging from 128 to 142 genes, primarily influenced by IR expansion/contraction events and pseudogenization of ndhF, ndhI, and ndhG. Two large inversions were detected within the large single‐copy region, including a synapomorphic inversion encompassing 19 genes. Phylogenomic analyses of 74 species confirmed the monophyly of Daphne but indicated paraphyletic for other genera.

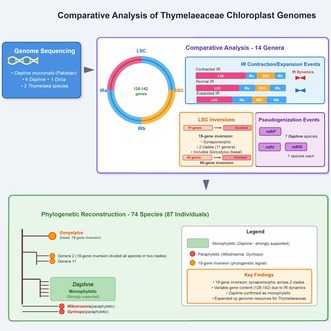

**Abbreviations**
CDS: coding sequence
cp: chloroplast
IR: inverted repeat
IRa/IRb: inverted repeat A/B
ITS: internal transcribed spacer
JLA: junction between IRa and LSC
JLB: junction between LSC and IRb
JSA: junction between SSC and IRa
JSB: junction between IRb and SSC
LSC: large single‐copy region
NCBI: National Center for Biotechnology Information
SSC: small single‐copy region
SSR: simple sequence repeat

The family Thymelaeaceae Juss., comprises approximately 52 genera and around 987 species, distributed across temperate and tropical regions worldwide [1]. It is divided into two subfamilies: Octolepidoideae and Thymelaeoideae. The former includes genera such as *Octolepis* Oliv. and *Gonystylus* Teijsm. & Binn., whereas the latter encompasses the tribes Aquilarieae, Daphneae and Synandrodaphneae [2]. Despite advances in molecular phylogenetics, taxonomic inconsistencies persist within Thymelaeaceae, even when analyses are based on complete chloroplast (cp) genome sequences. Such discrepancies are evident in genera such as *Daphne* Tourn. ex L., *Edgeworthia* Meisn., *Wikstroemia* Endl. and *Stellera* L. [3, 4, 5].

The genus *Daphne*, one of the largest within the family, comprises approximately 95 species, many of which are valued for their ornamental appeal and are widely distributed across Eurasia and North Africa [2, 6]. Beyond horticultural uses, *Daphne* species are known for their medicinal properties. Over 350 secondary metabolites have been identified from various species, exhibiting a broad spectrum of biological activities including antifungal, antibacterial, antiviral, antioxidant, anti‐inflammatory, cytotoxic, analgesic, abortifacient and hemostatic effects [7].

Despite its significance, the phylogenetic placement of *Daphne* remains controversial. Most species form a clade with the sister genus *Thymelaea* Mill., suggesting a close evolutionary relationship. However, some species exhibit affinities with other genera and are embedded within their clades, rendering *Daphne* paraphyletic. Notably, the distinction between *Daphne* and *Wikstroemia* Endl. remains unresolved, with ongoing debate as to whether these should be maintained as separate genera or merged based on morphological and molecular evidence [3, 4]. Species such as *Daphne championii* Benth. (grouping with *Edgeworthia*), *Daphne genkwa* Siebold & Zucc. and *Daphne aurantiaca* Diels (grouping with *Wikstroemia*) deviate from traditional classifications. These cases highlight the need for taxonomic revision based on both morphological characteristics and molecular data [3, 4]. A recent study proposed reclassifying *D. aurantiaca* and *D. genkwa* into *Wikstroemia*, citing their morphological features, cp genome characteristics and phylogenetic positions [8]. Similarly, *Stellera* has been shown to form a sister relationship with *Wikstroemia*, further complicating generic boundaries [3, 4, 8]. Although Zhang *et al*. [8] did not rule out the possible inclusion of *Stellera chamaejasme* within *Wikstroemia*, they maintained that, under current taxonomic frameworks, *Stellera* remains a distinct genus as a result of its long‐standing taxonomic recognition. Nevertheless, their phylogenetic analyses revealed that *Wikstroemia* is not monophyletic and shares a closer evolutionary relationship with *Stellera* than with *Daphne*.

Advancements in high‐throughput sequencing have facilitated comprehensive investigations of nuclear, mitochondrial and cp genomes, significantly contributing to the fields of evolutionary biology, phylogenetics, population genetics and natural product research [9, 10, 11, 12, 13]. The cp genome, typically a circular DNA molecule, exhibits a conserved quadripartite structure consisting of a large single‐copy (LSC) region, a small single‐copy (SSC) region and two inverted repeat (IR) regions [11, 14, 15]. It serves as a suitable molecular marker as a result of various mutational events, including insertions and deletions (indels), nucleotide substitutions, IR expansions and contractions, and inversions [16, 17, 18]. Although cp genomes are generally conserved in terms of gene content and arrangement [16, 19], significant variation has been reported within Thymelaeaceae, including IR boundary shifts, gene loss and pseudogenization [3, 4, 5, 8]. For example, *Wikstroemia nutans* Champ. ex Benth. has lost the *ndhA*, *ndhG* and *ndhI* genes, whereas other genes, such as *ndhD*, *ndhE*, *ndhH*, *psaC*, *ccsA*, *rps15* and *trnL‐UAG*, occur as single copies as a result of IR contraction [8]. Similarly, *D. genkwa*, recently proposed for transfer to *Wikstroemia*, possesses one of the shortest cp genomes (~132 kb) and displays extensive gene loss and pseudogenization, including *ndhA*, *ndhI*, *ndhF*, *rpl32*, *ndhH*, *ndhG* and *ndhD*. Its unique IR boundary configuration also results in the conversion of formerly duplicated genes into single‐copy genes [4, 20].

Previous comparative analyses of cp genomes in Thymelaeaceae have been relatively limited in scope. For example, one study focused exclusively on eight species of *Daphne* [4]. Likewise, studies on *Aquilaria* Lam. [21], combined analyses of *Aquilaria* and *Gyrinops* Gaertn. [22] and investigations of *Wikstroemia* [5, 8] were restricted to genus‐level comparisons. These works primarily examined the cp genome structure and standard features, such as IR contraction and expansion, codon usage, amino acid frequency, and simple sequence repeat (SSR) identification at the genus level. Although valuable, none of these studies provided a comprehensive family‐level assessment of cp genome structure across multiple genera or identified larger‐scale structural rearrangements, such as inversions and IR boundary shifts, in a family‐wide context. Our study addresses these gaps by expanding taxon sampling across 14 genera, assembling additional cp genomes, and conducting a more comprehensive comparative analysis of cp genomes to explore inversion events and variations in IR contraction and expansion at the family level. In Pakistan, three species of *Daphne* are reported: *Daphne papyracea* Wall. ex G. Don, *Daphne retusa* Hemsl. and *Daphne mucronata* Royle. Among these, cp genome sequences for *D. papyracea* and *D. retusa* are already available in the National Center for Biotechnology Information (NCBI) database. In the present study, we collected *D. mucronata* from Pakistan, sequenced it and performed *de novo* assembly of its cp genome. Because no complete cp genome from the sister genus *Thymelaea* had previously been publicly available, we also *de novo* assembled the cp genomes of two *Thymelaea* species using publicly accessible raw sequencing data. Additionally, to expand the genomic resources within *Daphne*, we *de novo* assembled cp genomes for four more *Daphne* species (Table 1). The cp genome of *Dirca occidentalis* A. Gray was also assembled to further enrich the genomic representation of the family.

**Table 1:** Accession numbers, quantity of raw data and coverage depth of *de novo* assembled chloroplast genome.

Species	Accession no.	Data downloaded (GB)	Coverage depth	WGS reads (millions)	Chloroplast reads (millions)
*Daphne mucronata*	SRR31580145	15.23	462×	36.51	0.53
*Daphne blagayana*	ERR14050974	2.08	161×	6.82	0.27
*Daphne cneorum*	ERR14050964	4.20	256×	13.74	0.44
*Daphne petraea*	ERR14043046	4.02	237×	12.96	0.40
*Daphne mezereum*	ERR5554756	2.17	80×	8.41	0.14
*Dirca occidentalis*	SRR28374817	139.12	103×	333.67	0.12
*Thymelaea dioica*	ERR13996336	6.78	407×	22.25	0.71
*Thymelaea passerina*	ERR14050971	4.09	453×	13.41	0.78

In the present study, the *de novo* assembled cp genomes of eight species, comprising five *Daphne*, two *Thymelaea* and one *Dirca*, were compared with the cp genomes of previously reported species. These newly assembled genomes were analyzed alongside cp genomes of 12 additional *Daphne* species and 11 species representing other genera within Thymelaeaceae. In total, cp genomes from 17 *Daphne* species and representatives of 13 other genera were included in the comparative analysis. For phylogenetic reconstruction, a comprehensive dataset comprising 73 Thymelaeaceae species was utilized. The aims and objectives of this study were: (i) to expand genomic resources for *Daphne*, *Dirca* and *Thymelaea* by assembling their complete cp genomes, thereby contributing to the enrichment of cp genome data for the family Thymelaeaceae; (ii) to perform comparative analyses of cp genomes within *Daphne* to investigate genetic variation and structural differences among its species, and to further compare *Daphne* cp genomes with those of representative species from other genera in Thymelaeaceae, aiming to assess patterns of sequence divergence, structural variation and gene content at both the genus and family levels; and (iii) to reconstruct a robust phylogeny of Thymelaeaceae by integrating newly assembled and publicly available cp genome data, with particular emphasis on resolving the evolutionary placement of *Daphne* and its related genera.

## Materials and methods

### Plant collection, DNA extraction and sequencing

**Fig. 1:**
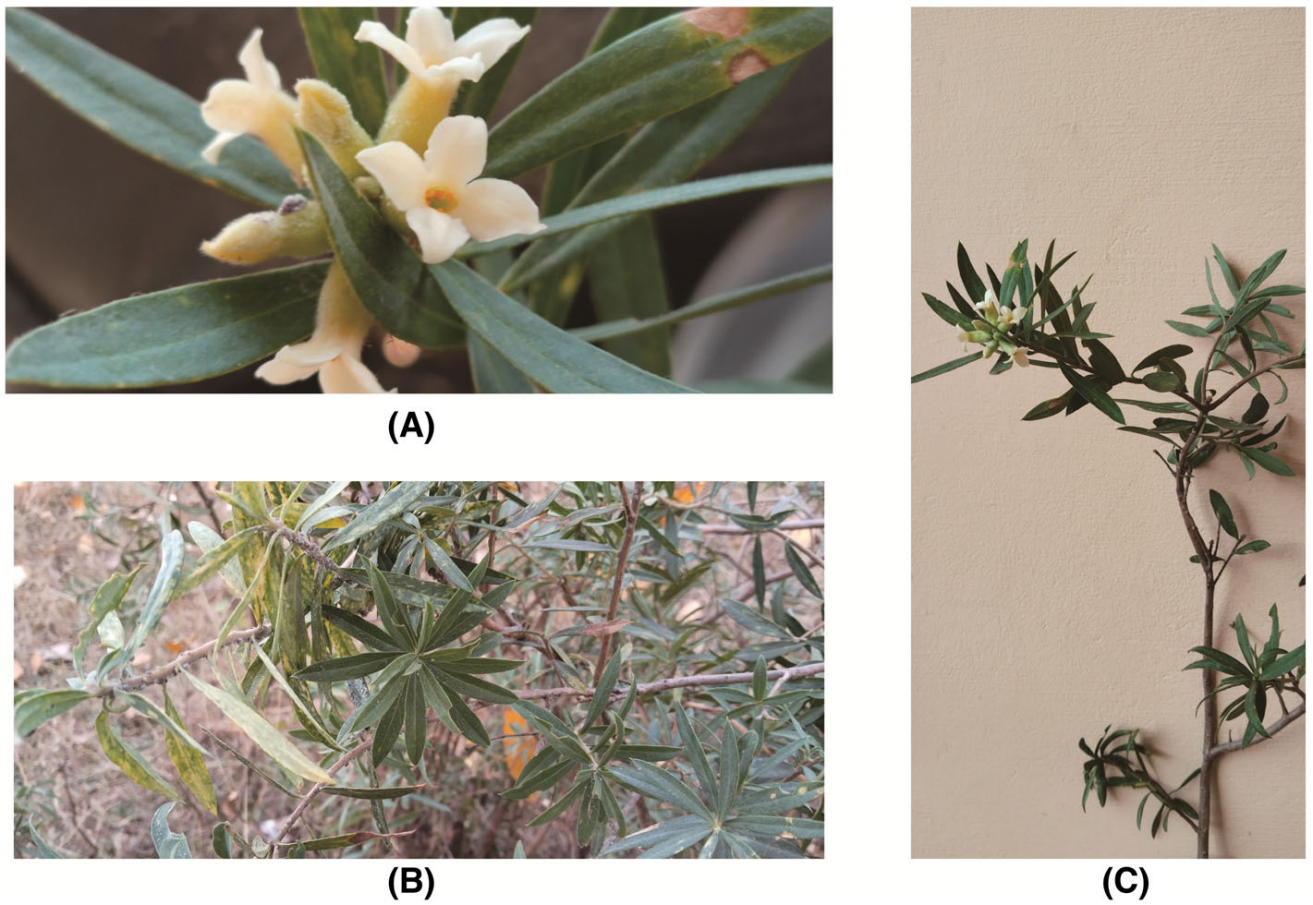
Photos of *Daphne mucronata*. (A) Close‐up of flowers highlighting reproductive structures. (B) Detailed view emphasizing leaf and stem morphology. (C) Overview of the entire plant, showing flowers, leaves and stems.

*D. mucronata* was collected from District Mansehra, Khyber Pakhtunkhwa, Pakistan (34°36′10″N, 73°23′20″E) (Fig. 1). The species was identified by Dr Abdul Majid (Department of Botany, Hazara University, Mansehra) and a voucher specimen was deposited in the university herbarium under accession number HUP16845. Representative photographs are provided in Fig. S1. Genomic DNA was extracted from silica‐dried leaves using the DNeasy Plant Mini Kit (Qiagen, Hilden, Germany), in accordance with the manufacturer's instructions. The extracted DNA was used for whole‐genome shotgun sequencing on the Illumina NovaSeq 6000 platform (Illumina, San Diego, CA, USA), generating 150‐bp paired‐end reads with an average insert size of 350 bp.

### *De novo* assembly and annotations of cp genomes

In total, eight cp genomes were *de novo* assembled in this study. For *D. mucronata*, raw sequencing reads were newly generated, whereas data for seven additional species, comprising *Daphne blagayana* Freyer (ERR14050974), *Daphne cneorum* L. (ERR14050964), *Daphne mezereum* L. (ERR5554756), *Daphne petraea* Leyb. (ERR14043046), *D. occidentalis* A. Gray (SRR28374817), *Thymelaea dioica* (Gouan) All. (ERR13996336) and *Thymelaea passerina* (L.) Coss. & Germ. (ERR14050971), were retrieved from the NCBI Sequence Read Archive (https://www.ncbi.nlm.nih.gov/sra) and the European Nucleotide Archive (https://www.ebi.ac.uk/ena). These species were selected to enhance taxonomic representation within *Daphne* and Thymelaeaceae and to generate the first cp genome assemblies for the genera *Thymelaea* and *Dirca*.

cp genomes were *de novo* assembled using getorganelle v1.7.5+ with default parameters [23]. Genome annotation was performed using geseq v2.03 [24], incorporating aragorn v.1.2.38 [25] and trnascan‐se v.2.0.7 [26] to annotate tRNAs. Start and stop codon inconsistencies were manually curated by initially aligning the assembled genomes with reference cp genomes of *Daphne depauperata* (NC_057149), *D. papyracea* (OP580924) and *Edgeworthia chrysantha* (MT135125) using geneious R8.1 [27]. Following annotation, all cp genomes were cross‐compared to minimize errors, and additional species used in comparative analyses were similarly checked to ensure annotation accuracy. Sequencing coverage depth was calculated by mapping raw reads of each species to its assembled cp genome under default settings in geneious R8.1. Genome maps were generated with chloroplot [28].

### Comparative genomics and phylogenetic analysis

The eight *de novo* assembled cp genomes were compared with those of 24 additional species, including 12 *Daphne* species and 11 species from other genera within Thymelaeaceae. To ensure annotation consistency, all cp genomes were reannotated because previous studies reported annotation errors in publicly available sequences [17, 29].

Comparative analyses were conducted in geneious R8.1 [27] to assess genome features, gene content and structural variation. Collinear block alignment was performed using Mauve progressive alignment [30] to assess gene order. IR boundary dynamics were visualized using cpjsdraw [31] to determine IR contraction/expansion, whereas nucleotide diversity across coding and non‐coding regions was quantified using cpstool [32].

The *ndhF* gene was identified as a pseudogene in seven *Daphne* species. To validate this, comparative alignments were conducted across other *Daphne* and Thymelaeaceae species. Codon usage and amino acid frequencies were analyzed using custom python scripts (Data S3 and S4). SSRs were identified using the misa‐web tool [33] with the minimum thresholds: 10 repeats for mononucleotides, five for dinucleotides, four for trinucleotides, and three for tetra‐, penta‐ and hexanucleotides. SSRs located within 100 bp of each other were considered compound SSRs.

In total, 87 individuals, representing 74 species of Thymelaeaceae, were included in the phylogenetic analysis. Some species were represented by multiple accessions, resulting in a total of 88 datasets. As a result of extensive IR contraction and expansion across the family, which has been associated with rate heterotachy [34, 35], only the coding sequence (CDS) shared across the LSC regions of all samples were extracted, and then the CDS of each species were concatenated using geneious. This strategy was adopted because several Thymelaeaceae species exhibit partial or complete loss of genes [4, 8], making it impractical to use the entire set of cp CDS without introducing significant bias because of missing data. Thus, focusing on the shared LSC‐region CDS ensured a more robust and reproducible phylogenetic reconstruction.

The concatenated CDS of each species were multiple aligned using mafft [36]. *Hibiscus mutabilis* (MK820657; Malvaceae) was used as the outgroup [18]. The final alignment comprised 4089 parsimony‐informative sites and 3257 distinct site patterns (Data S1). Phylogenetic reconstruction was performed using the maximum likelihood method in iq‐tree v3 [37]. modelfinder [38] identified the best‐fit nucleotide substitution model (GTR + F + I + R4) under the Bayesian information criterion. Branch support was assessed using ultrafast bootstrap approximation (ufboot) [39] and the Shimodaira–Hasegawa approximate likelihood ratio test, each with 10 000 replicates. To refine tree topology, the bootstrap‐guided Nearest Neighbor Interchange (−bnni) was employed. The analysis was run with automatic CPU thread detection (−nt AUTO) to maximize computational efficiency. The resulting phylogenetic tree was visualized and annotated using the Interactive Tree of Life (itol) platform [40].

## Results

### Chloroplast genomes assembly and coverage depth analysis

Sequencing of *D. mucronata* generated 15.23 GB of data (36.51 million reads), including 0.53 million cp‐specific reads, enabling cp genome assembly with a coverage depth of 462×. Additionally, cp genomes for seven other species were *de novo* assembled from publicly available sequencing reads in NCBI (Table 1). These datasets ranged from 2.08 GB (6.82 million reads) to 139.1 GB (333.7 million reads), yielding average coverage depths of 80× to 462× (Fig. S2). The number of cp‐specific reads for these assemblies ranged from 0.12 to 0.78 million (Table 1). These reads were found to be helpful for *de novo* assembly of all genomes in a circular form with high coverage depth without any misassemblies or chimeric sequences.

### Chlororoplast genomes feature and variations in total gene counts

The eight newly assembled cp genomes, representing five *Daphne*, two *Thymelaea* and one *Dirca* species, displayed genomic characteristics consistent with other members of Thymelaeaceae (Table 2). Except for *Dicranolepis disticha*, cp genome sizes across Thymelaeaceae ranged from 169 944 to 176 548 bp, with conserved structures comprising a LSC region (84158–88 950 bp), a SSC region (2179–3374 bp) and two Irs (41 406–43 948 bp) (Table 3). *D. disticha* had a smaller genome of 154 903 bp, with longer SSC and shortened IRs as a result of IR contraction. These genomes are provided as Data S2.

**Table 2:** Functional classification of genes in the chloroplast genome of *Daphne*. *Indicates genes containing introns. A superscript ‘a’ next to specific genes denote those duplicated in the IR regions. *ndhF* gene is a pseudogene in seven species (*Daphne acutiloba*, *Daphne depauperate*, *Daphne feddei*, *Daphne genkwa*, *Daphne kiusiana*, *Daphne* sp. TX‐2022a and *Daphne tangutica*).

Genes categories and groupings		Name of genes	Number
Self‐replication	Large subunit of ribosome	*rpl14*, rpl16*, rpl2*^,*a*^, *rpl20*, *rpl22*, *rpl23* ^*a*^, *rpl32*, *rpl33*, *rpl36*	11
	Small subunit of ribosome	*rps11*, rps12*, *rps14*, *rps15* ^*a*^, rps16*, *rps18*, *rps19*, *rps2*, *rps3*, *rps4*, *rps7* ^*a*^, *rps8*	14
	DNA dependent RNA polymerase	*rpoA*, *rpoB*, rpoC1*, *rpoC2*	4
	rRNA genes	*rrn16* ^*a*^, *rrn23* ^*a*^, *rrn4.5* ^*a*^, *rrn5* ^*a*^	8
	tRNA genes	trnA‐UGC*^,*a*^, *trnC‐GCA*, *trnD‐GUC*, *trnE‐UUC*, *trnF‐GAA*, *trnG‐GCC*, trnG‐UCC*, *trnH‐GUG*, *trnI‐CAU* ^*a*^, trnI‐GAU*^,*a*^, trnK‐UUU*, *trnL‐CAA* ^*a*^, trnL‐UAA*, *trnL‐UAG* ^*a*^, *trnM‐CAU*, *trnN‐GUU* ^*a*^, *trnP‐UGG*, *trnQ‐UUG*, *trnR‐ACG* ^*a*^, *trnR‐UCU*, *trnS‐GCU*, *trnS‐GGA*, *trnS‐UGA*, *trnT‐GGU*, *trnT‐UGU*, *trnV‐GAC* ^*a*^, trnV‐UAC*, *trnW‐CCA*, *trnY‐GUA*, *trnfM‐CAU*	38
Photosynthesis	Photosystem I	*psaA*, *psaB*, *psaC* ^*a*^, *psaI*, *psaJ*	6
	Photosystem II	*psbA*, *psbB*, *psbC*, *psbD*, *psbE*, *psbF*, *psbH*, *psbI*, *psbJ*, *psbK*, *psbL*, *psbM*, *psbN*, *psbT*, *psbZ*	15
	NADPH dehydrogenase	ndhA*^,*a*^, ndhB*^,*a*^, *ndhC*, *ndhD* ^*a*^, *ndhE* ^*a*^, *ndhF*, *ndhG* ^*a*^, *ndhH* ^*a*^, *ndhI* ^*a*^, *ndhJ*, *ndhK*	18
	Cytochrome *b*/*f* complex	*petA*, petB*, petD*, *petG*, *petL*, *petN*	6
	Subunits of ATP synthase	*atpA*, *atpB*, *atpE*, atpF*, *atpH*, *atpI*	6
	Large subunit of RuBisCO	*rbcL*	1
Other genes	Maturase	*matK*	1
	Envelop membrane protein	*cemA*	1
	Acetyl‐CoA‐carboxylase subunit	*accD*	1
	C‐type cytochrome synthesis gene	*ccsA* ^*a*^	2
	Protease	*clpP*	1
	Conserved open reading frames	*ycf1* ^*a*^, *ycf2* ^*a*^, ycf3*, *ycf4*	6
Total number of genes			139

**Table 3:** Comparative analysis of chloroplast genome among 33 species of the family Thymelaeaceae.

Species	Length (bp)				GC content (%)				GC content of genes (%)			
	Total	LSC	SSC	IR	Total	LSC	SSC	IR	tRNA	rRNA	CDS	Accession
*Aquilaria sinensis*	174 914	87 361	3347	42 103	36.71	34.94	29.04	38.85	53.37	55.56	37.23	MN720647
*Daphne acutiloba*	171 211	85 136	2179	41 948	36.73	34.8	29.92	38.87	52.93	55.45	37.33	NC_056317
*Daphne mezereum*	170 915	84 690	2855	41 685	36.52	34.63	28.14	38.81	52.93	55.47	37.2	BK070978
*Daphne blagayana*	171 748	85 189	2879	41 840	36.71	34.82	28.69	38.9	52.93	55.49	37.32	BK071447
*Daphne cneorum*	171 088	84 487	2771	41 915	36.75	34.88	28.91	38.89	52.86	55.47	37.31	BK071448
*Daphne depauperata*	170 916	84 812	2276	41 914	36.73	34.81	28.69	38.89	52.93	55.47	37.33	NC_057149
*Daphne feddei*	171 084	84 837	2463	41 892	36.73	34.8	28.79	38.91	52.93	55.47	37.33	NC_056320
*Daphne giraldii*	171 643	85 171	2876	41 798	36.75	34.86	28.62	38.96	52.9	55.47	37.36	NC_044085
*Daphne kiusiana*	171 491	85 028	2681	41 891	36.71	34.79	28.35	38.92	52.93	55.47	37.32	NC_035896
*Daphne laureola*	171 613	85 316	2855	41 721	36.72	34.86	28.97	38.88	52.97	55.47	37.36	MN201546
*Daphne mucronata*	171 190	84 507	2822	41 930	36.76	34.92	29.2	38.87	52.9	55.47	37.32	PQ858248
*Daphne odora*	171 156	84 681	2707	41 884	36.72	34.81	29	38.91	52.93	55.47	37.3	MT627479
*Daphne papyracea*	171 450	85 027	2705	41 859	36.72	34.81	28.8	38.91	52.93	55.47	37.3	OP580924
*Daphne petraea*	171 298	84 709	2841	41 874	36.75	34.88	28.9	38.91	52.93	55.47	37.35	BK071449
*Daphne pseudomezereum* var. *koreana*	171 152	84 963	2739	41 725	36.52	34.59	28.55	38.74	52.97	55.4	37.2	ON244034
*Daphne retusa*	170 553	84 886	2437	41 615	36.75	34.83	28.19	38.96	52.93	55.47	37.34	MW245832
*Daphne tangutica*	169 944	84 884	2248	41 406	36.75	34.83	28.6	38.94	52.93	55.47	37.37	NC_042950
*Daphne championii*	174 773	84 158	2719	43 948	36.75	34.93	29.24	38.72	53.07	55.39	37.42	MT648376
*Diarthron linifolium*	172 644	86 158	2859	41 813	36.8	34.97	29.31	38.93	53	55.45	37.35	MW566785
*Dicranolepis disticha*	154 903	85 215	19 194	25 247	37.41	35.16	32.1	43.23	52.47	55.58	38.11	MZ901896
*Dirca occidentalis*	173 021	86 227	2684	42 055	36.5	34.66	29.47	38.62	53.12	55.45	37.2	BK071450
*Edgeworthia chrysantha*	172 446	85 527	2871	42 024	36.6	34.8	29.7	38.8	53.0	55.4	37.3	MT135125
*Gonystylus affinis*	176 548	88 950	3374	42 112	36.58	34.89	30.02	38.62	53.14	55.49	37.2	MN147872
*Gyrinops caudata*	174 798	87 270	3346	42 091	36.72	34.93	29.26	38.88	53.16	55.64	37.27	MZ145050
*Phaleria macrocarpa*	174 585	86 568	3049	42 484	36.7	34.88	29.35	38.82	53.02	55.32	37.45	MN147873
*Pimelea aquilonia*	172 364	85 576	2805	41 991	36.65	34.78	29.59	38.8	53.37	55.4	37.29	MH230106
*Rhamnoneuron balansae*	172 971	85 819	2900	42 126	36.77	34.93	29.38	38.9	52.97	55.45	37.48	ON183350
*Stellera chamaejasme*	173 381	86 769	2858	41 877	36.68	34.84	29.25	38.85	53	55.45	37.59	MK681211
*Thymelaea dioica*	172 648	85 835	2807	42 003	36.72	34.78	29.36	38.95	52.99	55.43	37.39	BK071451
*Thymelaea passerina*	171 885	85 602	2795	41 744	36.63	34.67	29.16	38.9	53.13	55.4	37.29	BK071452
*Wikstroemia alternifolia*	173 697	86 694	2857	42 073	36.64	34.75	29.51	38.83	53	55.47	37.38	MW073913

**Fig. 2:**
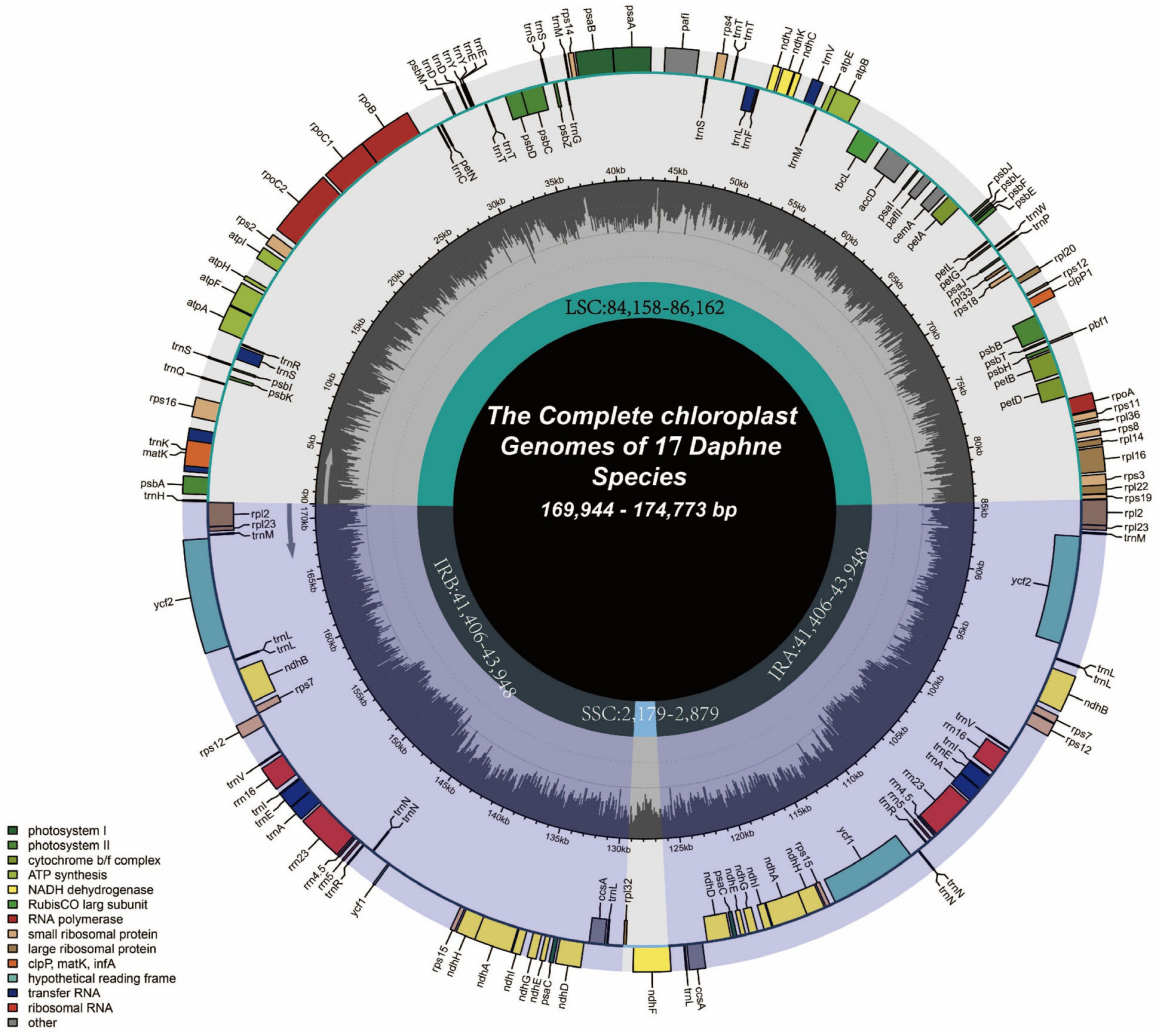
Chloroplast genome maps of *Daphne* species. Generalized cp genome structure representing *Daphne* species. Genes transcribed clockwise and counterclockwise are shown inside and outside the genome circle, respectively, and color‐coded based on their functional categories. The genome organization depicted in figure also illustrates the cp genome structure of the newly sequenced *Dirca* and *Thymelaea* species.

The cp genomes contained 110–112 unique genes, including 76–78 protein‐coding genes, four rRNA genes and 30 tRNA genes (Table 2). With the exception of *D. championii* and *D. disticha*, all species had 27 duplicated genes in the IRs (15 protein‐coding, four rRNA and eight tRNA) (Fig. 2 and Table 2). The trans‐spliced *rps12* gene was not included among the duplicated genes. Seventeen intron‐containing genes were identified, six of which were duplicated within the IR regions (Fig. 2 and Table 2).

Comparative analyses of 18 *Daphne* species and 13 representatives from other genera revealed differences in gene content. These genomes are also available as Data S2. *D. championii* contained 142 genes, which is three genes more than other species, as a result of duplications of *rps19*, *rpl3* and *rpl22*, which were present as single‐copy genes in other species. These duplications resulted in an increase from 15 to 18 duplicated CDS in *D. championii*.

*D. disticha* exhibited a distinct genome architecture as a result of IR contraction and expansion, which shifted 11 genes (*trnL‐UAG*, *ccsA*, *ndhD*, *ndhE*, *ndhG*, *ndhI*, *ndhA*, *ndhH*, *psaC*, *rps15* and *ycf1*) into single‐copy regions. By contrast, these were typically duplicated in other species. As a result, *D. disticha* had only 128 genes in total. These structural differences are discussed further below.

The overall GC content ranged from 36.52% to 36.77%. Region‐specific GC contents were 34.59–34.93% in the LSC, 28.19–29.92% in the SSC and 38.72–38.96% in the IRs. The elevated GC content of the IRs is attributable to rRNA genes, which reached up to 55% GC content.

### Pseudogenization of ndh genes

Pseudogenization of *ndhF* was observed in seven *Daphne* species, including *D. acutiloba*, *D. retusa*, *D. depauperata*, *Daphne feddei*, *Daphne kiusiana*, *Daphne* sp. *TX‐2022a* and *Daphne tangutica*. In addition, *ndhK* in *Daphne odora*, *ndhI* in *D. tangutica* and *ndhG* in *D. retusa* were also found as pseudogenes. In these species, *ndhF* either contained internal stop codons or existed as truncated fragments, as revealed by comparative alignments (Figs S3 and S4).

### Analysis of inversion events

**Fig. 3:**
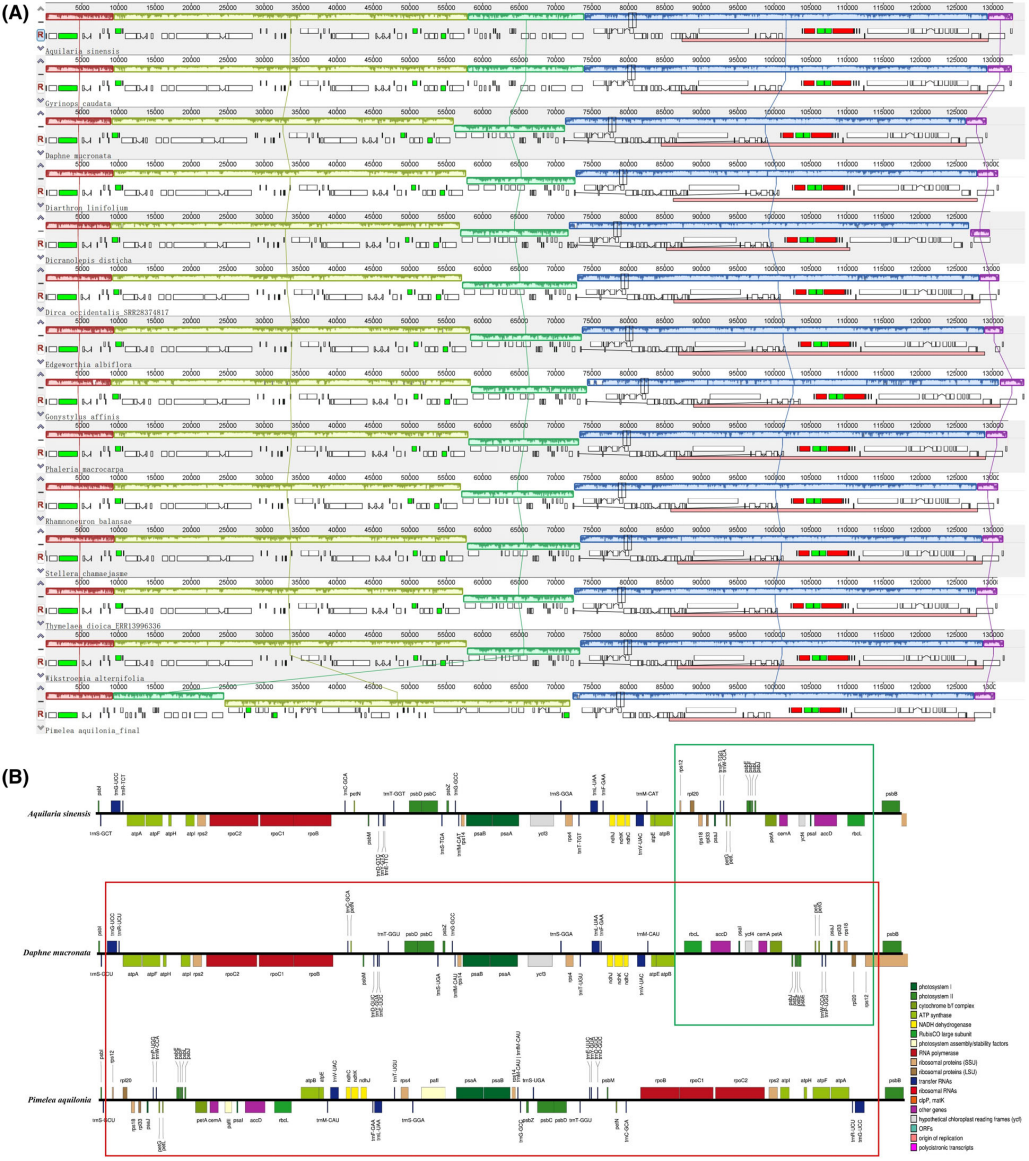
Comparative genomic analysis revealing two inversion events within Thymelaeaceae. (A) Mauve alignment of chloroplast genomes from 14 species, each representing one genus, highlighting two major inversion events indicated by yellow and green blocks. (B) Illustrating the impact of these inversions on gene arrangement, represented by one selected species from each of the three resulting groups (Group I, Group II and Group III). The green box indicates one type of inversion event, whereas the red box represents the other.

Mauve progressive alignment revealed two inversion events in the LSC region of cp genomes, using *D. mucronata* as the reference (Fig 3A,B). All species at the genus level displayed a similar gene order in the cp genome (Fig. S5), suggesting that the observed rearrangements are authentic structural features rather than assembly artifacts.

A unique inversion event was detected in *Pimelea aquilonia* Rye, distinguishing it from all other genera included in the analysis. Because *P. aquilonia* was the sole representative of its genus in this study, additional sampling will be necessary to determine whether this inversion is genus‐specific, species‐specific, or the result of an assembly artifact.

Based on the inversion patterns, the cp genomes of Thymelaeaceae species were grouped into three structural categories: Group I included *Aquilaria sinensis* and *Gyrinops caudata* (Gilg) Domke; Group II comprised 11 species: *D. mucronata*, *Diarthron linifolium* Turcz., *D. disticha*, *D. occidentalis*, *Edgeworthia albiflora* Nakai, *Gonystylus affinis* Radlk., *Phaleria macrocarpa* (Scheff.) Boerl., *Rhamnoneuron balansae* (Maury) Gilg, *S. chamaejasme* L., *Thymelaea dioica* and *Wikstroemia alternifolia* Batalin.; Group III consisted solely of *P. aquilonia*. The species in Group I formed a distinct phylogenetic group, all sharing a common cp structural type. Likewise, the species in Group II formed a separate group, defined by a different structural type. These inversion types thus represent synapomorphic characters that unite each group and reinforce their phylogenetic placement. *G. affinis* was positioned at the basal node from which both Groups I and II diverged, supporting the view that structural rearrangements are consistent with evolutionary divergence within the family. By contrast, *P. aquilonia* deviated from this general pattern, as it was grouped within the lineage of Group II but displayed unique long inversion events, indicating a lineage‐specific structural rearrangement. Please refer to the phylogenetic section for this grouping. The first inversion rearranged 19 genes: *rbcL*, *accD*, *psaI*, *ycf4*, *cemA*, *petA*, *psbJ*, *psbL*, *psbF*, *psbE*, *petL*, *petG*, *trnW*, *trnP*, *psaJ*, *rpl33*, *rps18*, *rpl20* and *rps12*. The second inversion affected 40 genes, including *pafII*, *psaI*, *atpB*, *atpE*, *trnM*, *trnV*, *ndhC*, *ndhK*, *ndhJ*, *trnF*, *trnL*, *trnT*, *rps4*, *trnS*, *psaA*, *psaB*, *rps14*, *trnM*, *trnG*, *psbZ*, *trnS*, *psbC*, *psbD*, *trnT*, *trnE*, *trnY*, *trnD*, *psbM*, *petN*, *trnC*, *rpoB*, *rpoC1*, *rpoC2*, *rps2*, *atpI*, *atpH*, *atpF*, *atpA*, *trnR* and *trnG* (Fig. 3A,B).

### Inverted repeats contraction and expansion

**Fig. 4:**
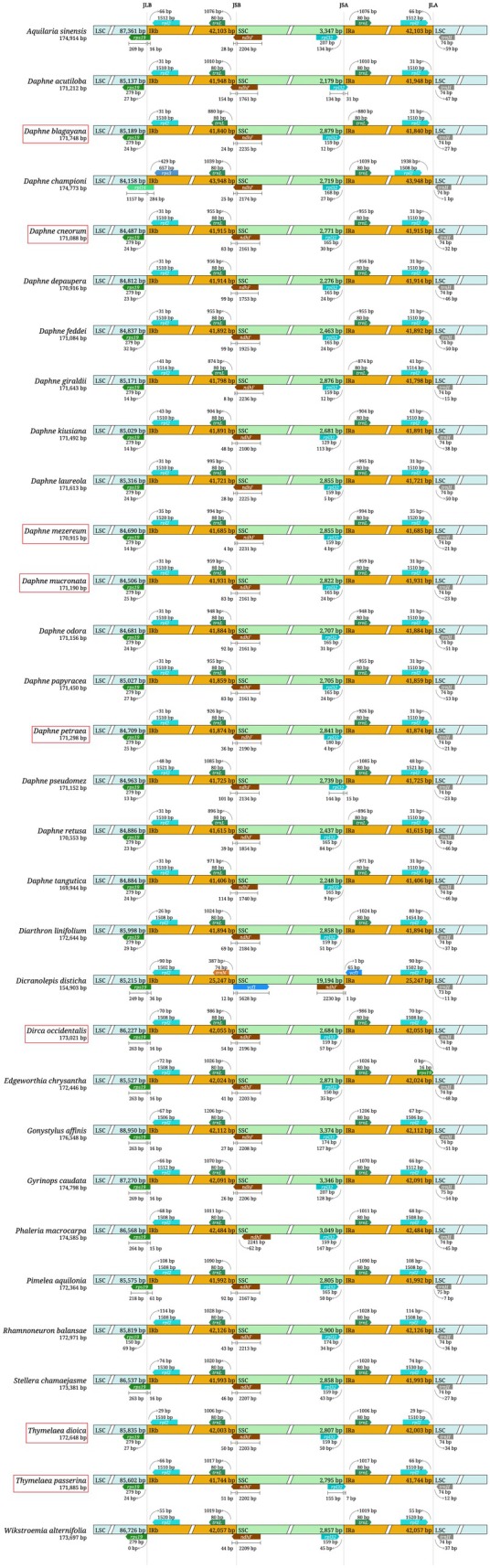
Comparison of inverted repeat expansions and contractions among 33 Thymelaeaceae species. Red boxes indicate species with cp genomes newly assembled in this study. Junction sites are abbreviated as: JLB (LSC/IRb), JSB (IRb/SSC), JSA (SSC/IRa) and JLA (IRa/LSC).

Except for *D. championii* and *D. disticha*, all species exhibited conserved gene arrangements at the four junction regions: JLB (LSC/IRb), JSB (IRb/SSC), JSA (SSC/IRa) and JLA (IRa/LSC) (Fig. 4). These species in total contained 138–139 genes, including 92–93 CDS, eight rRNA genes and 38 tRNA genes. Specifically, *rps19* and *rpl2* were located at the JLB junction, *trnL* and *ndhF* at JSB, *rpl32* and *trnL* at JSA, and *rpl2* and *trnH* at JLA.

*D. disticha* differed in gene placement at certain junctions. *rps19* and *rpl2* were located at JLB, *trnN* and *ycf1* at JSB, *ndhF* and *trnN* at JSA, and *rpl2* and *trnH* at JLA. Unlike other species, which contained 138–139 genes, *D. disticha* contained only 128 genes. This difference resulted from the presence of 11 genes (*trnL‐UAG*, *ccsA*, *ndhD*, *ndhE*, *ndhG*, *ndhI*, *ndhA*, *ndhH*, *psaC*, *rps15* and *ycf1*) in the single‐copy region instead of the IR regions.

In *D. championii*, *rpl16* and *rps3* were located at the JLB junction, whereas the other junctions were similar to those of most species. IR expansion in *D. championii* led to the transfer of *rps3*, *rpl22* and *rps19* from the LSC region into the IR regions, where they are now duplicated. These genes were present as single copies in the other species. This species contained a total of 142 genes, including 96 protein‐coding genes. The distinct IR contraction and expansion patterns observed in these two species were not correlated with their phylogenetic placement.

### Codon usage and amino acid frequency analysis

Relative synonymous codon usage analysis across *Daphne* species identified a pronounced bias toward codons terminating in adenine (A) or thymine (T).

Codons ending in A/T consistently exhibited relative synonymous codon usage values ≥ 1, reflecting higher usage frequencies, whereas those ending in cytosine (C) or guanine (G) showed values ≤ 1 (Table S1). This trend suggests a preference for the use of A/T‐rich codons during translation. Amino acid composition analysis further revealed high conservation among species, with lysine and isoleucine being the most abundant residues (Table S2). By contrast, cysteine was the least represented amino acid, suggesting limited utilization in protein sequences across the genus.

### SSR analyses

The analysis of SSRs across 18 *Daphne* species revealed that mononucleotide repeats were the most abundant type, ranging from 57 in *D. mucronata* to 83 in both *D. blagayana* and *D. mezereum* (Tables S3 and S4). Dinucleotide repeats showed relatively stable frequencies across species, typically numbering between 9 and 13 (Table S3). Tri‐, tetra, penta‐ and hexanucleotide repeats were less frequent, with hexanucleotide SSRs being rare, detected in only two species (*D. cneorum* and *Daphne pseudomezereum*). Overall, SSR counts varied among species, with the highest observed in *D. mezereum* (108) and the lowest in *D. mucronata* (78), highlighting species‐specific variation in SSR content. Most of these SSR motifs consisted of A/T instead of C/G (Table S4).

### Nucleotide diversity analysis of *Daphne* cp genome regions

**Fig. 5:**
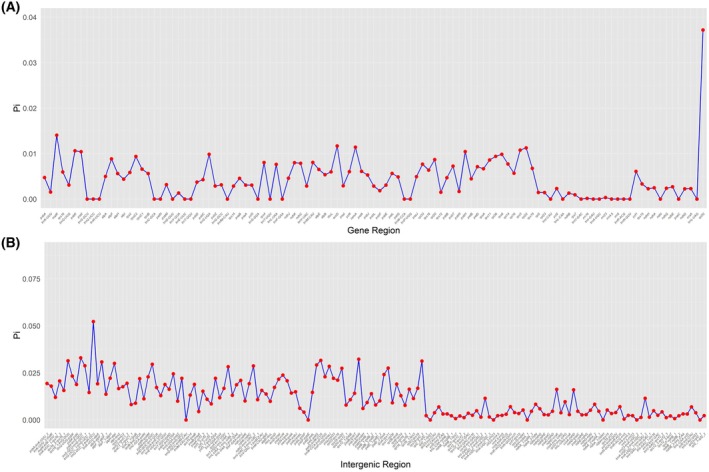
Nucleotide diversity across different regions of the cp genome in *Daphne* species. (A) Nucleotide diversity of protein‐coding, transfer RNA and ribosomal RNA genes. (B) Nucleotide diversity of intergenic spacers and introns.

In this study, we compared the genomic regions of *Daphne* species and identified five highly polymorphic loci from protein‐coding sequences: *rpl32*, *matK*, *accD*, *cemA* and *rpl22*. Additionally, we identified five polymorphic loci from intergenic spacer regions: *trnG‐UCC‐trnR‐UCU*, *trnH‐GUG‐psbA*, *psbI‐trnS‐GCU*, *psbT‐psbN* and *petG‐trnW‐CCA* (Fig. 5). Detailed information is presented in Table 4. The nucleotide diversity of protein‐coding regions ranged from 0.01 to 0.05, whereas that of intergenic spacer regions ranged from 0.02 to 0.05.

**Table 4:** Identification of suitable polymorphic loci based on comparative analysis of *Daphne* species.

Serial number	Region	Nucleotide diversity	Number of substitutions	Number of indels	Region length	Alignment length	Missing data (%)
1	*rpl32*	0.037	41	2	159	183	13.1
2	*matK*	0.014	148	2	1517	1527	0.7
3	*accD*	0.012	115	6	1524	1617	5.7
4	*cemA*	0.011	57	0	696	696	0
5	*rpl22*	0.011	42	0	504	504	0
6	*rps3*	0.011	47	1	657	660	0.5
7	*psbK*	0.010	16	0	186	186	0
8	*psbH*	0.010	18	1	222	228	2.6
9	*psbI*	0.010	5	0	111	111	0
10	*rps8*	0.010	24	0	405	405	0
11	*trnG‐UCC‐trnR‐UCU*	0.052	18	1	58	180	67.8
12	*trnH‐GUG‐psbA*	0.041	63	14	270	410	34.1
13	*psbI‐trnS‐GCU*	0.033	13	6	74	91	18.7
14	*psbT‐psbN*	0.032	6	1	70	82	14.6
15	*petG‐trnW‐CCA*	0.032	29	2	147	180	18.3
16	*rps16‐trnQ‐UUG*	0.031	97	26	426	1506	71.7
17	*rps19‐rpl2*	0.031	11	1	48	63	23.8
18	*atpA‐atpF*	0.031	15	3	74	77	3.9
19	*atpH‐atpI*	0.030	120	23	708	1276	44.5
20	*psbM‐trnD‐GUC*	0.030	44	7	283	823	65.6

### Phylogenetic analysis

**Fig. 6:**
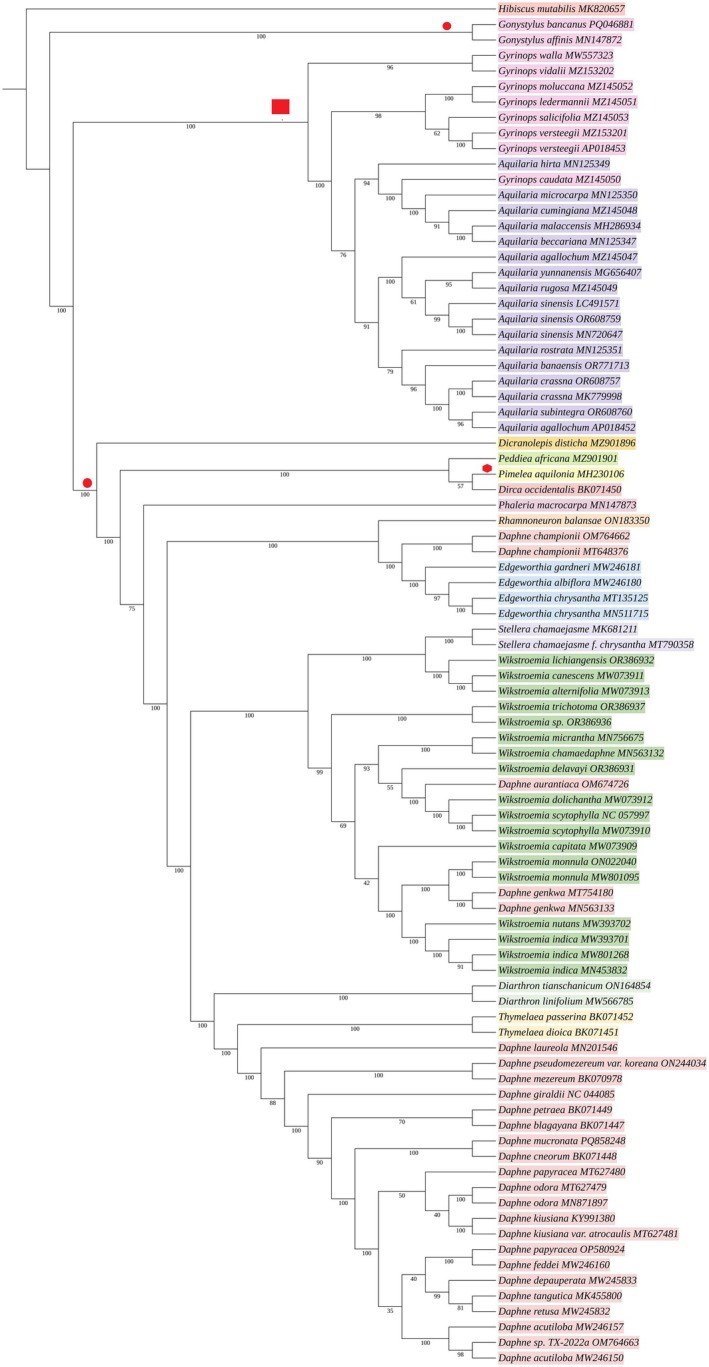
Maximum‐likelihood phylogenetic tree inferred from 73 species across 14 genera of Thymelaeaceae using iq‐tree v3. Bootstrap support values are shown at each node. Background colors group species belonging to the same genus. Red shapes placed at specific nodes mark gene inversion events: circular and square boxes each represent a distinct inversion type involving the same set of 19 genes, with all species descending from that node sharing the same arrangement. The octagonal shape denotes a unique inversion involving 40 genes, observed only in *Pimelea aquilonia*. These inversion patterns highlight the synapomorphic nature of the events shared by the two main clades.

The robust phylogenetic analysis of 74 species (87 individuals) representing 14 genera provided valuable insights into the evolutionary relationships within Thymelaeaceae (Fig. 6). The analysis recovered a strongly supported, monophyletic *Daphne* clade comprising 17 species, which collectively formed a sister relationship with *Thymelaea*. *D. championii* was resolved as sister to *Edgeworthia*, whereas *D. aurantiaca* and *D. genkwa*, both of which have recently been proposed for transfer to *Wikstroemia*, were nested within the *Wikstroemia* clade. Among other genera, *G. caudata* was embedded within *Aquilaria*, and *Stellera* formed a sister relationship with *Wikstroemia*. The newly assembled *Daphne* species, comprising *D. mucronata*, *D. blagayana*, *D. cneorum*, *D. mezereum* and *D. petraea*, grouped with other members of *Daphne* within the monophyletic clade and did not exhibit paraphyly. Additionally, the *de novo* assembled *D. occidentalis* was resolved as sister to *P. aquilonia*.

## Discussion

The comparative analysis in this study demonstrates a largely conserved cp genome structure within the genus *Daphne*, although notable exceptions were found. Comparative genomics further highlighted structural variations and gene loss events or pseudogenization across the family Thymelaeaceae.

### Conserved nature of the cp genome of *Daphne* species

The cp genome exhibits a combination of conserved and polymorphic features across plant lineages. In *Daphne* species, the cp genomes retain the typical quadripartite structure, comprising an LSC, SSC and two Irs, consistent with patterns reported in other angiosperm families such as Asteraceae, Solanaceae and Malvaceae [17, 29, 41]. This structural conservation across divergent lineages highlights the evolutionary constraints imposed by the cp genome's essential roles in photosynthesis and gene regulation [42].

Despite this overall conservation, variability was observed in gene content. The *ndhF* gene was pseudogenized in seven *Daphne* species, *ndhK* in *Daphne odora*, *ndhI* in *Daphne tangutica* and *ndhG* in *Daphne retusa*, suggesting relaxed functional constraints or lineage‐specific mutations. Pseudogenization of *ndh* genes is not uncommon in plants and has been associated with shifts in ecological niches or photosynthetic adaptations [43]. Because these findings are based on reported genomes, we were unable to verify them through raw read mapping or independent validation using Sanger sequencing because of the unavailability of material. Therefore, additional validation will be required to establish the authenticity and evolutionary significance of these pseudogenization events.

### cp genome structure variation across Thymelaeaceae, inverted repeats contraction and expansion, and its link to phylogeny

cp genomes across the Thymelaeaceae family display largely conserved gene content, consistent with previous studies on this family and other angiosperms [4, 21, 41, 44]. However, structural divergences such as genome rearrangements and shifts in IR boundaries highlight underlying genomic plasticity. For example, two major inversions relocated 19 and 40 genes without disrupting their functionality. Although rare in plants, such rearrangements have been reported in Cyperaceae and Fabaceae, suggesting that cp genomes can tolerate positional gene shifts at the same time as maintaining functional integrity, possibly reflecting their roles in environmental adaptation [19, 45, 46]. These inversions may result from recombination events mediated by repetitive sequences, a phenomenon documented in other lineages with dynamic cp genomes [47]. The first inversion, distinguishing Groups I and II (Fig. 3), was consistently observed across all 73 analyzed species, underscoring its evolutionary significance. Group I, comprising 23 species from two genera, exhibited a unique gene arrangement distinct from the 50 species across 11 genera in Group II. This widespread conservation of a synapomorphic inversion across divergent taxa strongly supports its authenticity as a structural feature rather than an assembly artifact. It also aligns with documented patterns in plant lineages where structural rearrangements contribute to speciation and adaptive divergence [45, 46, 48]. The second inversion, involving 40 genes, was unique to *P. aquilonia* (Group III), which, based on its phylogenetic position, was embedded within the species of Group II. Although its singularity raises questions regarding its evolutionary origin, comparable rare rearrangements in other taxa have occasionally been attributed to lineage‐specific adaptations or neutral drift [46]. Nevertheless, the possibility of an assembly artifact cannot be entirely excluded without further validation. Future studies incorporating additional sequencing data, broader taxon sampling and long‐read genome assemblies are therefore required to confirm the stability and evolutionary implications of this inversion.

Notable IR expansions and contractions were observed in *D. championii* and *D. disticha*. In *D. championii*, IR expansion duplicated *rps19*, *rps3* and *rpl22*, mirroring patterns seen in *Abelmoschus esculentus* (Malvaceae) [41], whereas other IR junctions remained conserved across *Daphne* species. Conversely, *D. disticha* exhibited pronounced structural variation, including IR boundary shifts and gene rearrangements resembling those in Malvaceae (Malvales) [18, 41, 49]. These parallels may reflect shared evolutionary pressures or lineage‐specific genomic instability, although broader taxonomic sampling is necessary to confirm this.

Intriguingly, IR boundary alterations in Thymelaeaceae did not exhibit a clear correlation with phylogenetic relationships, reflecting similar patterns reported in Araceae [35, 50]. By contrast, several other plant groups have demonstrated strong associations between IR boundary dynamics and their phylogenetic or population structures, highlighting the taxon‐specific nature of these genomic changes [16, 51].

### Identification of polymorphic loci as molecular markers in *Daphne*

The utility of cp genomic regions for species identification and phylogenetic inference depends largely on their variability, necessitating careful selection of highly polymorphic markers [52, 53]. In this study, 10 hypervariable regions were identified across *Daphne* species, including five protein‐coding genes (*rpl32*, *matK*, *accD*, *cemA* and *rpl22*) and five intergenic spacers (*trnG‐UCC–trnR‐UCU*, *trnH‐GUG–psbA*, *psbI–trnS‐GCU* and *psbT–psbN*, *petG–trnW‐CCA*). These regions exhibited nucleotide diversity values ranging from 0.01 to 0.05 in genes and 0.02 to 0.05 in intergenic‐spacers, highlighting their potential as robust molecular markers for resolving taxonomic uncertainties and reconstructing interspecific relationships within *Daphne*.

Notably, our results differ from a previous study that identified *psaI* and the *ndhF‐rpl32* spacer as polymorphic loci [4]. These discrepancies likely arise from methodological differences: we used cpstool to assess nucleotide diversity across the entire cp genome with data from 18 species, whereas the earlier study employed a sliding window approach in DnaSP based on only six species. Such methodological factors, including sample size and analytical tools, can significantly influence marker selection because broader taxonomic sampling enhances the detection of variable regions and reduces sampling bias [54]. Smaller datasets may miss loci exhibiting lineage‐specific variability, underscoring the importance of comprehensive species representation in marker discovery [55].

### Phylogenetic insights and implications for Thymelaeaceae classification

The phylogenetic analysis based on cp genome data from 74 species (87 individuals) strongly supports previous findings regarding relationships within *Daphne* [3, 4, 8]. Notably, *D. aurantiaca* and *D. genkwa* grouped with species of the genus *Wikstroemia*, whereas *D. championii* formed a sister group to *Edgeworthia*, consistent with earlier studies. These findings reinforce the need for taxonomic reassessment of these genera. Recently, *D. aurantiaca* and *D. genkwa* were proposed for transfer to *Wikstroemia* [8]. Prior studies based on morphological and molecular analyses also suggested reclassifying certain species, including *D. genkwa*, at the same time as emphasizing that *Daphne* and *Wikstroemia* should generally be maintained as separate genera. These studies identified a polyphyletic position of some species based on plastome and nuclear internal transcribed spacer (ITS) phylogenies and highlighted morphological features, such as the opposite leaf arrangement of *D. genkwa*, rare among *Daphne*, and its divided floral disks, which are typical of *Wikstroemia* [3, 4]. The sister relationship between *D. championii* and *Edgeworthia*, inferred from plastome and nuclear ITS markers, further supports previous suggestions to exclude *D. championii* from *Daphne* because this species exhibits long styles, elongated filaments and short upright calyx teeth, distinguishing it from typical *Daphne* species [4]. We agree with this view; however, further taxonomic and morphological investigations are required to determine whether *D. championii* should be transferred to an existing genus or maintained as a distinct evolutionary lineage.

Among other genera analyzed, *G. caudata* was embedded within the *Aquilaria* clade and *S. chamaejasme* showed a sister relationship with *Wikstroemia*. These results are consistent with previous studies [5, 22]. Although *G. caudata* may potentially be transferred to *Aquilaria*, this hypothesis requires further evaluation through broader taxon sampling. The present dataset includes 14 of the 21 recognized *Aquilaria* species and seven of the nine *Gyrinops* species, supporting their current recognition as distinct genera. Nevertheless, the close affinity of *G. caudata* with *Aquilaria* supports prior suggestions of its possible reassignment [22]. The sister relationship between *Stellera* and *Wikstroemia* also remains taxonomically complex. Although Zhang *et al*. [8] acknowledged the potential inclusion of *S. chamaejasme* within *Wikstroemia*, they recommended retaining *Stellera* as a distinct genus because of its long‐standing taxonomic status and morphological distinctiveness. Notably, the newly assembled *Daphne* species, comprising *D. mucronata*, *D. blagayana*, *D. cneorum* and *D. petraea*, clustered within a well‐supported *Daphne* clade, showing no evidence of paraphyly.

Overall, these findings underscore the need for a comprehensive phylogenetic framework for Thymelaeaceae. Future studies should incorporate broader species sampling and higher‐resolution approaches, such as transcriptome sequencing or multilocus nuclear data generated through low‐coverage whole genome sequencing or target capture of nuclear genes (e.g. Angiosperms353), as successfully applied in other plant lineages such as Asteraceae [56, 57, 58].

## Conclusions

In the present study, we *de novo* assembled the chloroplast genomes of eight species and conducted comparative analyses with previously published genomes of the Thymelaeaceae family. Our results revealed two major inversion events in the LSC region, identified pseudogenization of genes and documented variation in gene content driven by contraction and expansion of the IR regions. Additionally, phylogenetic analyses reinforced earlier evidence of paraphyly in certain genera, underscoring the need for taxonomic revision within Thymelaeaceae. These findings provide important insights into the structural evolution of chloroplast genomes and the phylogenetic complexity of the family.

## Conflicts of interest

The authors declare that they have no conflicts of interest.

## Author contributions

AM and A were responsible for plant collection and identification. A, HL and CC were responsible for writing the original draft. A, MI, RY, HL, AM and CC were responsible for data analysis and data curation. A, MI, PH and XT were responsible for data interpretation. A, IA, PH and XT were responsible for conceptualization. A, IA, PH and XT were responsible for reviewing and editing the manuscript.

## Supporting information


**Fig. S1.** *Daphne mucronata* in natural habitat and herbarium specimen.

**Fig. S2.** Coverage depth analysis of the eight *de novo* assembled chloroplast genomes performed in geneious.

**Fig. S3.** Multiple sequence alignment of the *ndhF* gene among *Daphne* species, highlighting pseudogenization events.

**Fig. S4.** Enlarged view of the *ndhF* gene alignment among *Daphne* species, showing detailed evidence of structural changes such as premature stop codons and insertion–deletion (indel) events.

**Fig. S5.** Mauve alignment of chloroplast genomes from species representing nine genera of *Thymelaeaceae*, illustrating gene arrangement conservation at the genus level.

**Table S1.** Relative synonymous codon usage analysis among *Daphne* species.

**Table S2.** Comparison of amino acid frequency among *Daphne* species.

**Table S3.** Comparison of SSR numbers and their types among *Daphne* species.

**Table S4.** Simple sequence repeats motifs and their types in species of *Daphne*.

**Data S1.** Final aligned CDS matrix used for phylogenetic analysis (FASTA format).

**Data S2.** Annotated chloroplast genome sequences of all newly assembled species (GenBank format).

**Data S3.** Python scirpt for analysis of codon usage.

**Data S4.** Python script for analysis of amino acid frequency.

## Data Availability

The complete cp genome sequence of *D. mucronata* is available under PQ858248, whereas the raw reads of sequencing data are available under BioProject PRJNA1193258.
