# Supplementary material for: Evolutionary dynamics of the chloroplast genome in Daphne (Thymelaeaceae): comparative analysis with related genera and insights into phylogenetics
Source: FEBS Open Bio. 2025 Oct 16;16(3):503–19. doi: 10.1002/2211-5463.70143 (PMC12955755; doi:10.1002/2211-5463.70143)
Supplement: Supplementary file 1 — Fig. S1. Daphne mucronata in natural habitat and herbarium specimen. [file FEB4-16-503-s012.pdf]

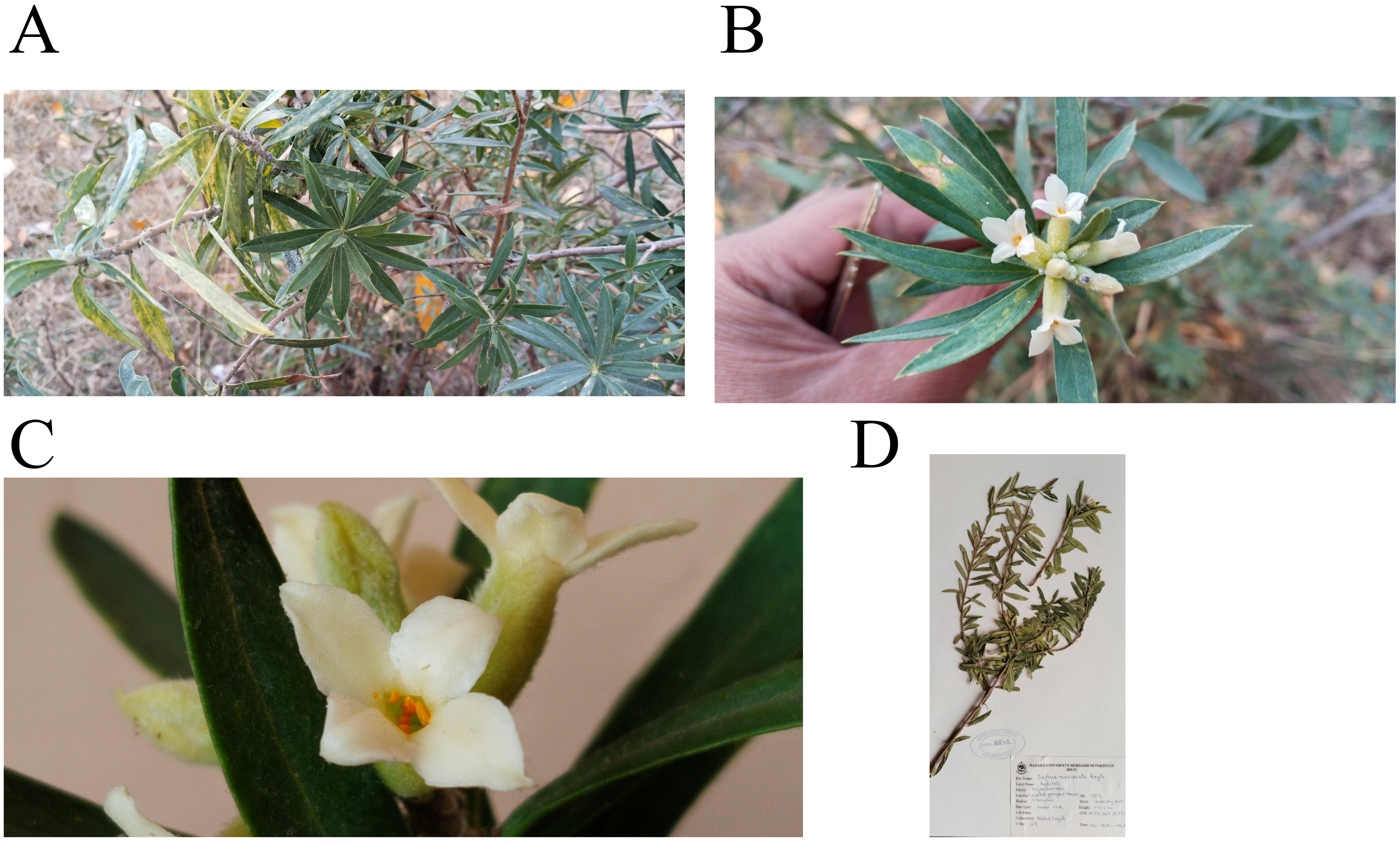

Figure S1. *Daphne mucronata* in natural habitat and herbarium specimen. (A) The leaves, stems, and branches of the plant in its natural environment. (B) The arrangement of flowers on the plant. (C) A close-up view of a single flower. (D) The herbarium specimen of *D. mucronata*.
