# Supplementary figures and images for: Evolutionary dynamics of the chloroplast genome in Daphne (Thymelaeaceae): comparative analysis with related genera and insights into phylogenetics
Source: FEBS Open Bio. 2025 Oct 16;16(3):503–19. doi: 10.1002/2211-5463.70143 (PMC12955755; doi:10.1002/2211-5463.70143)

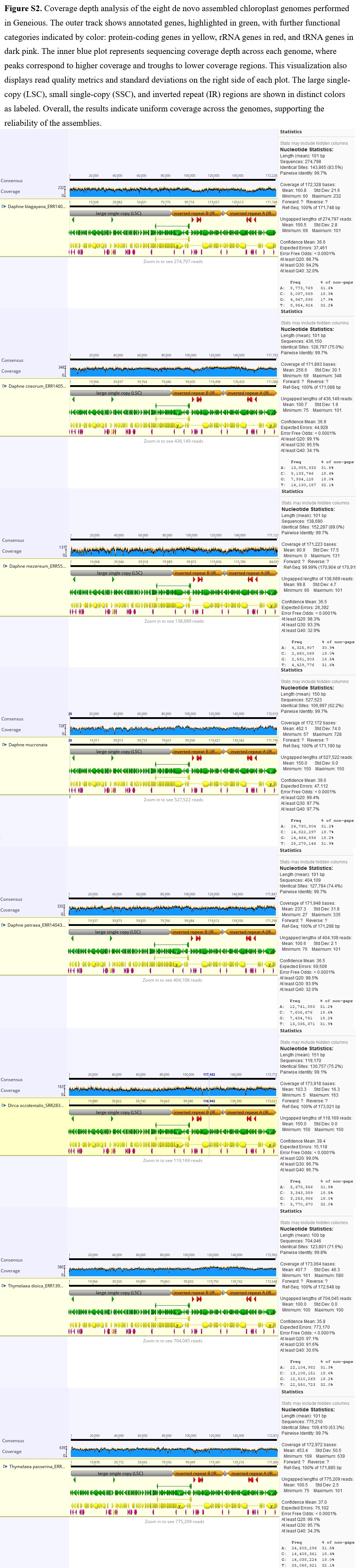

Supplement: Supplementary file 2 — Fig. S2. Coverage depth analysis of the eight de novo assembled chloroplast genomes performed in geneious. [file FEB4-16-503-s010.jpg]
