# Supplementary material for: Evolutionary dynamics of the chloroplast genome in Daphne (Thymelaeaceae): comparative analysis with related genera and insights into phylogenetics
Source: FEBS Open Bio. 2025 Oct 16;16(3):503–19. doi: 10.1002/2211-5463.70143 (PMC12955755; doi:10.1002/2211-5463.70143)

Figure S3. Multiple sequence alignment of the *ndhF* gene among *Daphne* species, highlighting pseudogenization events. Premature stop codons and frameshift mutations indicating pseudogenization are marked with black asterisks on the yellow CDS track. The upper green line, containing multiple colors, represents nucleotide variation across the alignment, where breaks or changes in the line indicate mutations. A thick black line denotes conserved regions of the gene, whereas thinner segments indicate indels and deleted sequences. Genes are shown in dark green, while CDS regions are highlighted in yellow. This visualization enables comparison of sequence integrity across species and identifies the specific mutations responsible for pseudogenization in some *Daphne* taxa.

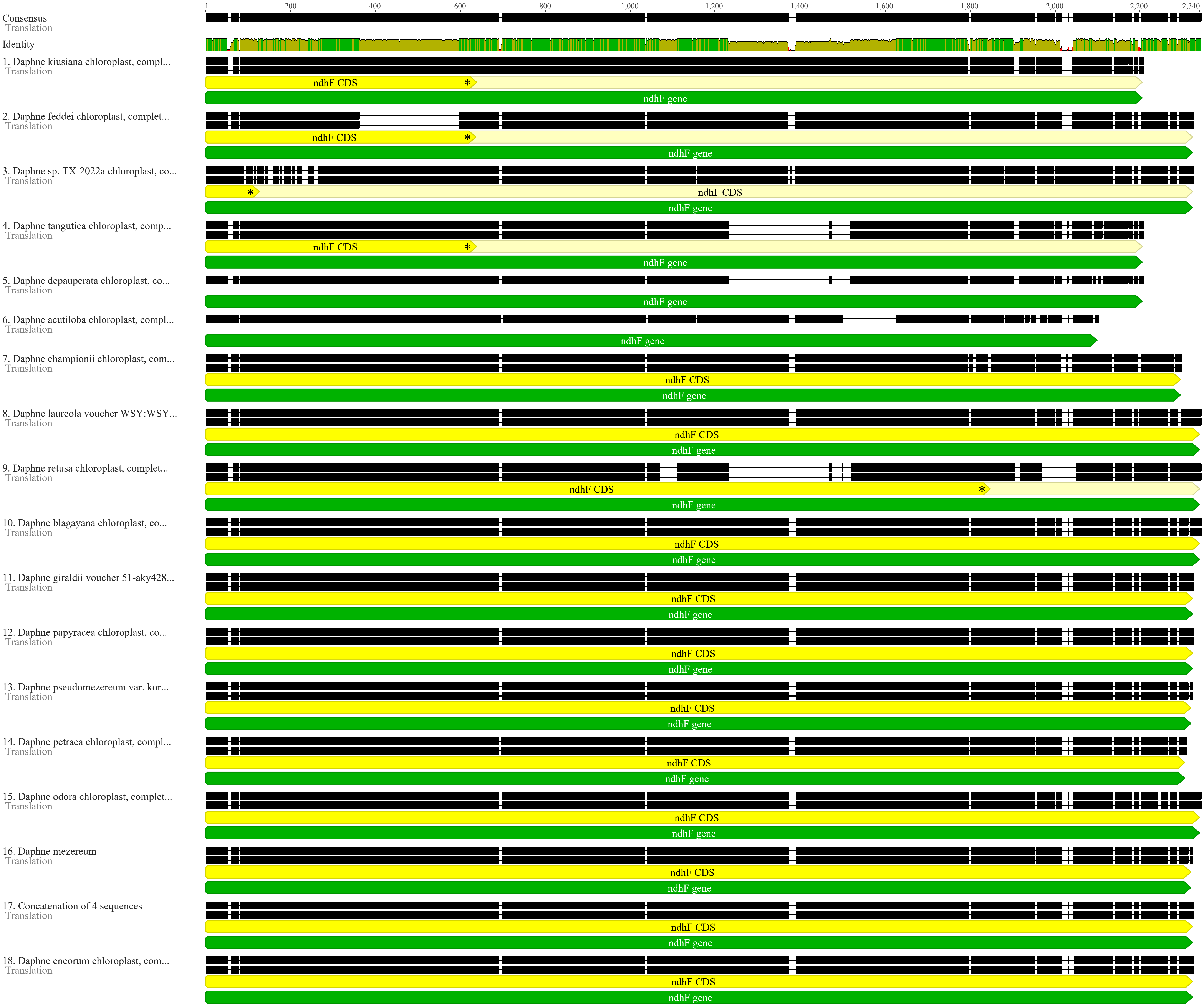

Supplement: Supplementary file 3 — Fig. S3. Multiple sequence alignment of the ndhF gene among Daphne species, highlighting pseudogenization events. [file FEB4-16-503-s004.pdf]
